# Effect of replacing dicalcium phosphate with mono-dicalcium phosphate to supplement phosphorus on laying performance, phosphorus-calcium metabolism and bone metabolism of aged laying hens

**DOI:** 10.3389/fvets.2023.1196334

**Published:** 2023-06-02

**Authors:** Yuechang Ren, Tianyu Zhao, Kaiying Zhang, Zhengqi Zhu, Linkui Li, Yang Li, Shuzhen Jiang, Ning Jiao, Weiren Yang

**Affiliations:** ^1^Department of Animal Sciences and Technology, Shandong Agricultural University, Tai’an, China; ^2^College of Food Science and Engineering, Shandong Agricultural University, Tai’an, China; ^3^Laiyang Animal Husbandry and Veterinary Bureau, Yantai, China

**Keywords:** aged laying hen, egg quality, bone metabolism, phytase, mono-dicalcium phosphate, phosphorus-calcium metabolism

## Abstract

The objective of this study was to evaluate the effect of replacing dicalcium phosphate (DCP) with mono-dicalcium phosphate (MDCP) to formulate low-phosphorus (P) diets on laying performance, egg quality, phosphorus-calcium metabolism, and bone metabolism of 69–78-week-old aged laying hens. Hy-Line Brown laying hens (*n* = 1,350, 69 weeks old) were randomly assigned to six treatments, each with five replicates of 45 hens. A corn–soybean meal–based diet was formulated to contain 0.12% non-phytate phosphorus (NPP), 3.81% calcium (Ca), and 1,470 FTU/kg phytase. The control group (CON) was supplemented with DCP inorganic phosphorus (Pi) at the NPP level of 0.20% (dietary NPP levels of 0.32%). Test groups (T1–T5) were supplemented with MDCP Pi at NPP levels of 0.07%, 0.11%, 0.15%, 0.18, and 0.20% (dietary NPP levels of 0.19, 0.23, 0.27, 0.30, and 0.32%, respectively). Calcium carbonate levels were adjusted to ensure all experimental diets contained the same Ca levels (3.81%). The feeding trial lasted 10 weeks, with hens increasing in age from 69 to 78 weeks. When supplemented with 1,470 FTU/kg phytase, extra DCP Pi or MDCP Pi did not affect (*p* > 0.05) laying performance (day laying rate, average egg weight, feed intake, feed-to-egg mass ratio, broken egg rate), egg quality (eggshell strength, albumen height, haugh units), or serum P, Ca, copper (Cu), iron (Fe), zinc (Zn), and manganese (Mn) levels. However, when laying hens were fed MDCP Pi (NPP levels of 0.07 to 0.20%), yolk color improved (*p* = 0.0148). The tibia breaking strength was significantly higher (*p* < 0.05) in the 0.18 and 0.20% NPP MDCP Pi groups than in the 0.20% NPP DCP Pi group. The breaking strength, Ca content, and P content of tibia in 0.11% and 0.15% NPP MDCP Pi hens were not significantly (*p* > 0.05) different from those in 0.20% NPP DCP Pi hens. Hens fed 0.07% NPP MDCP Pi had higher (*p* < 0.01) serum levels of osteoprotegerin (OPG), type-I collagen c-telopeptide (CTX-I), and tartrate-resistant acid phosphatase 5b (TRACP-5b) than those in all other groups. Serum levels of TRACP-5b and CTX-I in the 0.11% and 0.15% NPP MDCP Pi group were significantly lower than those in 0.18 and 0.20% NPP MDCP Pi groups and the 0.20% NPP DCP Pi group (*p* < 0.0001). Hens fed 0.07% and 0.11% NPP MDCP Pi had higher (*p* < 0.05) serum levels of parathyroid hormone (PTH) than those in all other groups. No differences were detected in serum calcitonin (CT), 1,25-dihydroxy-vitamin D3 (1,25-(OH)2D3), bone alkaline phosphatase (BAP), osteocalcin(OCN), and osteopontin (OPN) among all groups (*p* > 0.05). The expression of P transporters type IIa Na/Pi cotransporter (NaPi-IIa) in 0.11% and 0.15% NPP MDCP Pi hens were higher than those in 0.20% NPP MDCP Pi group and 0.20% NPP DCP Pi group (*p* < 0.05). The results indicated that both renal P reabsorption and bone resorption were involved in adapting to a low-P diet. In summary, when MDCP was used instead of DCP to supplement P, NPP levels could be reduced to 0.11% (dietary NPP level of 0.23%) without negative effects on laying performance and skeletal health of aged hens. In addition, MDCP was more beneficial than DCP for tibia quality. The results of the current study would provide references for the application of MDCP in low-P diets of aged laying hens.

## Introduction

Phosphorus (P) is an essential nutrient for animal growth and development that has a crucial role in skeletal development and bone mineralization (1). However, more than two-thirds of the P in plant-based raw materials is phytate P. Monogastric animals, including poultry, cannot use bound P in phytate efficiently because of insufficient levels of endogenous phytase (2). Therefore, inorganic phosphates (Pi) such as mono-dicalcium phosphate (MDCP) and dicalcium phosphate (DCP) are commonly used in diets to support animal health and performance (3). However, P digestibility by poultry varies with different sources of Pi (3). Sauvant et al. (4) reported actual ileal P digestibility of MDCP and DCP produced from rock phosphate was 69.3 and 60.2%, respectively. According to Bikker et al. (5), actual ileal phosphate digestibility of DCP was 59.0%, whereas that of MDCP reached 70.7%. An et al. (6) reported apparent ileal digestibility of P in MDCP and DCP by poultry was 86.0 and 76.2%, respectively. Previous studies indicate that actual phosphate digestibility was lower for DCP from rock phosphates than for MDCP. Differences in Pi digestibility are attributed to phosphate characteristics as well as animal age, Ca:P ratio, and application of phytase (7, 8). Therefore, in-depth exploration of different mineral sources of P is required to adjust dietary requirements and ensure highly efficient production while reducing pressure on the environment.

In addition, phytase has been widely used in poultry diets to reduce Pi supplementation. Phytase is one of the most popular environmentally friendly feed additives of the 21st century. Supplementing with large amounts of phytase can further improve P utilization (9). Keshavarz (10) reported that supplementation with 300 FTU/kg phytase effectively restores laying performance when dietary NPP content is <0.25% at laying hens from 18 to 51 weeks of age. Wang et al. (11) indicated that laying performance could be maintained ifphytase at 360 FTU/kg was supplemented into a basal diet with supplementation of <0.10% NPP. Taheri et al. (12) and Adeola and Cowieson. (13) reported that dietary supplementation with phytase above 1,000 FTU/kg could reduce the amount of Pi, even without extra supplementation. Ren et al. (14) reported that corn–soybean meal-based diets containing 0.12% NPP and 2000 FTU/kg phytase would meet the requirements for egg production in Hy-Line Brown laying hens from 29 to 40 wk. of age. It is necessary to decrease dietary Pi supplementation because phosphate ore resources are limited globally and fecal P contaminates soils (15). Based on our understanding of the current literature, information is scant regarding the formulation of low-P diets for aged laying hens by replacing DCP with MDCP. The objective of this study was to evaluate the effect of replacing DCP with MDCP to formulate low-P diets on laying performance, egg quality, calcium-phosphorus metabolism, and bone metabolism of 69–78-week-old aged laying hens.

## Materials and methods

The Animal Care and Use Committee of Shandong Agriculture University (protocol code SDAUA-2021-019) approved this study.

### Animals and diets

A corn–soybean meal-based diet was formulated to contain 0.12% NPP, 3.81% Ca, and 1470 FTU/kg phytase (Table 1). Hy-Line Brown laying hens (*n* = 1350, 69 weeks old) were randomly assigned to six treatments, each with five replicates of 45 hens. The hens were housed in an environmentally controlled, three-tier, high-rise cage (depth × width × height = 47 × 36 × 105 cm) system. Three hens in the each of cage. The control group (CON) was supplemented with DCP at the NPP levels of 0.20% (dietary NPP levels of 0.32%), which is the current NPP level commonly set by Chinese premix companies when using phytase. Test groups (T1–T5) were supplemented with MDCP at NPP levels of 0.07%, 0.11%, 0.15%, 0.18%, and 0.20% (dietary NPP levels of 0.19%, 0.23%, 0.27%, 0.30%, and 0.32%, respectively). The MDCP was purchased from Yunnan Phosphate Group Co., Ltd (Kunming, China; Total *p* ≥ 21%), and the DCP was purchased from Sichuan Longmang Group Co., Ltd (Mianzhu, China; Total *p* ≥ 16.5%). Phytase (≥50,000 FTU/g; Ucommercial name: Habio phytase; 6-phytase produced by Escherichia coli) was purchased from Jinan Bestzyme Bio-engineering (Jinan, China). Calcium carbonate levels were adjusted to ensure all experimental diets contained the same level of Ca (3.81%). The trial was conducted under appropriate management to minimize discomfort to laying hens. The temperature in the laying room was maintained between 20.0°C and 25.0°C. Hens were provided with 16 h of light per day and had free access to feed and water throughout the experiment. The feeding trial was conducted for 10 weeks. The P sources and P levels of experimental diets are shown in Table 2. Laying performance, phosphorus-calcium metabolism, and bone metabolism were evaluated in Hy-Line Brown laying hens from 69 to 78 weeks of age.

**Table 1:** Composition and nutrient contents of the basal diet.

Item	% (except energy)
Corn	60.5
Soybean meal, 46%	22
Soybean oil	1
Calcium carbonate	8.5
Palmmeal	5
DCP	–
MDCP	–
Premix^a^	3
Total	100
Nutrient contents	
Analzed nutrient level (except energy)	
Metabolic energy (kcal/kg)	2,537
Crude protein	15.79
Calcium	3.81
Total phosphorus (analyzed)	0.34
NPP	0.12
Lysine	0.81
Methionine	0.42

a The premix provided the following per kilogram of diet: Vitamin A, 8100 IU; Vitamin D3, 2,700 IU; Vitamin E, 24 mg; Vitamin K3, 3.0 mg; Vitamin B1, 2.4 mg; Vitamin B2, 6 mg; Vitamin B6, 3 mg; Niacin, 10 mg; Folic acid, 1.2 mg; Pantothenic, 7.8 mg; VB12, 0.003 mg; Copper (from CuSO4.5H2O), 10.5 mg; Manganese (from MnSO4.H2O), 84 mg; Zinc (from ZnSO4.6H2O), 78 mg; Iron (from FeSO4.6H2O), 54.0 mg; Selenium (from Na2SeO3.5H2O), 0.5 mg; Iodine (from KT), 0.45 mg; Lysine, 0.7 g; Methionine, 1.77 g; NaCl, 3 g; phytase, 29. 1 mg.

**Table 2:** The P sources and P levels of experimental diets.

Treatment	P sources	NPP supplementation levels %	Dietary NPP levels %	Phytase (FTU/kg)	Calcium %
1	DCP	0.20	0.32	1,470	3.81
2	MDCP	0.07	0.19	1,470	3.81
3	MDCP	0.11	0.23	1,470	3.81
4	MDCP	0.15	0.27	1,470	3.81
5	MDCP	0.18	0.30	1,470	3.81
6	MDCP	0.20	0.32	1,470	3.81

#### Laying performance and egg quality

Feed consumption, number of eggs and total egg weight were recorded daily to calculate feed intake and feed-to-egg mass ratio. After the end of the feeding trial, 10 eggs per replicate (50 eggs per treatment) were randomly collected to determine egg quality. Haugh units, yolk color, and albumen height were measured using an automatic egg quality analyzer (EMT-5200; Robotmation, Co., Ltd., Tokyo, Japan). Eggshell strength was tested using a strength gauge (EFG-0503; Robotmation Co., Ltd.).

#### Serum biochemistry

At the end of the feeding trial, 10 hens (two hens per replicate) were randomly selected from each group. Fasting blood was collected from the left wing vein using coagulation-promoting tubes at 8:00 and 10:00 AM. Serum samples were extracted by centrifugation at 3,500 rpm for 15 min and then stored at −20°C until analysis (16). Serum Ca and P concentrations were determined at Shandong Agricultural University (Taian, China) using a fully automatic biochemical analyzer (L-7020; Hitachi, Tokyo, Japan). Serum concentrations of copper (Cu), iron (Fe), zinc (Zn), and manganese (Mn) were analyzed using a flame atomic absorption spectrometer (Z-8200, Hitachi) according to the method described in Chen et al. (17).

#### Tibia quality

After blood collection, hens were euthanized by cervical dislocation for tibia and kidney samples. Right tibia breaking strength was determined using a three-point bending test with a microcomputer-controlled electronic universal mechanical test machine (YAW-5000F; Jinan Pilot Gold Group Co., Jinan, China). The support distance of the instrument was 40 mm, and the testing speed was 2 mm/min. Left tibia samples were defatted in anhydrous ethanol (degreasing time: 8 h) and then dried (drying temperature: 105°C) to determine defatted dry weight. Then, samples were ashed (600°C, 8 h), and Ca (EDTA titration method) and P (ammonium metavanadate colorimetric method) contents were determined as reported in the study by Ren et al. (18). Ash, Ca and P contents of the defatted and oven-dried tibia were calculated.

#### Determination of serum Ca and P metabolism-related hormones

Ca and P metabolism-related hormones, including CT, PTH, and 1,25-(OH)2D3 were determined with the relevant enzyme-linked immunosorbent assay (ELISA) kits (Jiangsu Meimian Industrial, Yancheng, China) according to previous studies by Ren et al. (19).

#### Determination of serum bone turnover markers

Serum bone formation markers, including BAP, OCN and OPG, were determined with the relevant ELISA kits (Jiangsu Meimian Industrial) according to previous studies by Ren et al. (19).

Serum bone resorption markers, including CTX-I, OPN, and TRACP-5b, were determined with the relevant ELISA kits (Jiangsu Meimian Industrial) according to previous studies by Ren et al. (19).

#### Real-time reverse transcription-quantitative polymerase chain reaction (RT-qPCR)

Kidney samples were snap-frozen in liquid nitrogen and stored at – 80°C. Primer sequences used for real-time PCR and the procedure to determine relative mRNA expression were according to previous publication (20–22). The β-actin gene was amplified in parallel as the internal control for gene normalization, and primer sequences are shown in Table 3. The 2^−∆∆Ct^ method was used to calculate relative mRNA expression in renal samples.

**Table 3:** Primers for real-time PCR analysis in samples from Hy-Line Brown laying hens (69 to 78 weeks of age).

Gene Primer Primer sequence (5′–3′)			Predicted size (bp)	Annealing temperature (°C)
Na Pi-IIa	upstream primer downstream primer	5′-TTGGCCAGCACCAGACTCAG-3′ 5′-GCAGTAGGCAAACGGGGTC-3′	118	60 60
	upstream primer	5′-CTGGGGCTCATCTGAAGGGT-3′		60
*β*-actin	downstream primer	5′-GGACGCTGGGATGATGTTCT-3′	308	60

#### Statistical analyses

All data were analyzed by using the general linear model (GLM) in SAS 9.4 (SAS Institute Inc., Cary, NC, United States). The experimental unit was the replicate for the analysis of laying performance, each egg for egg quality parameters, and each hens for all other variables. The differences among treatments were compared with Tukey’s multiple range tests. Data are the mean ± standard error. Significance was considered at *p* < 0.05.

## Results

### Laying performance and egg quality

**Figure 1:**
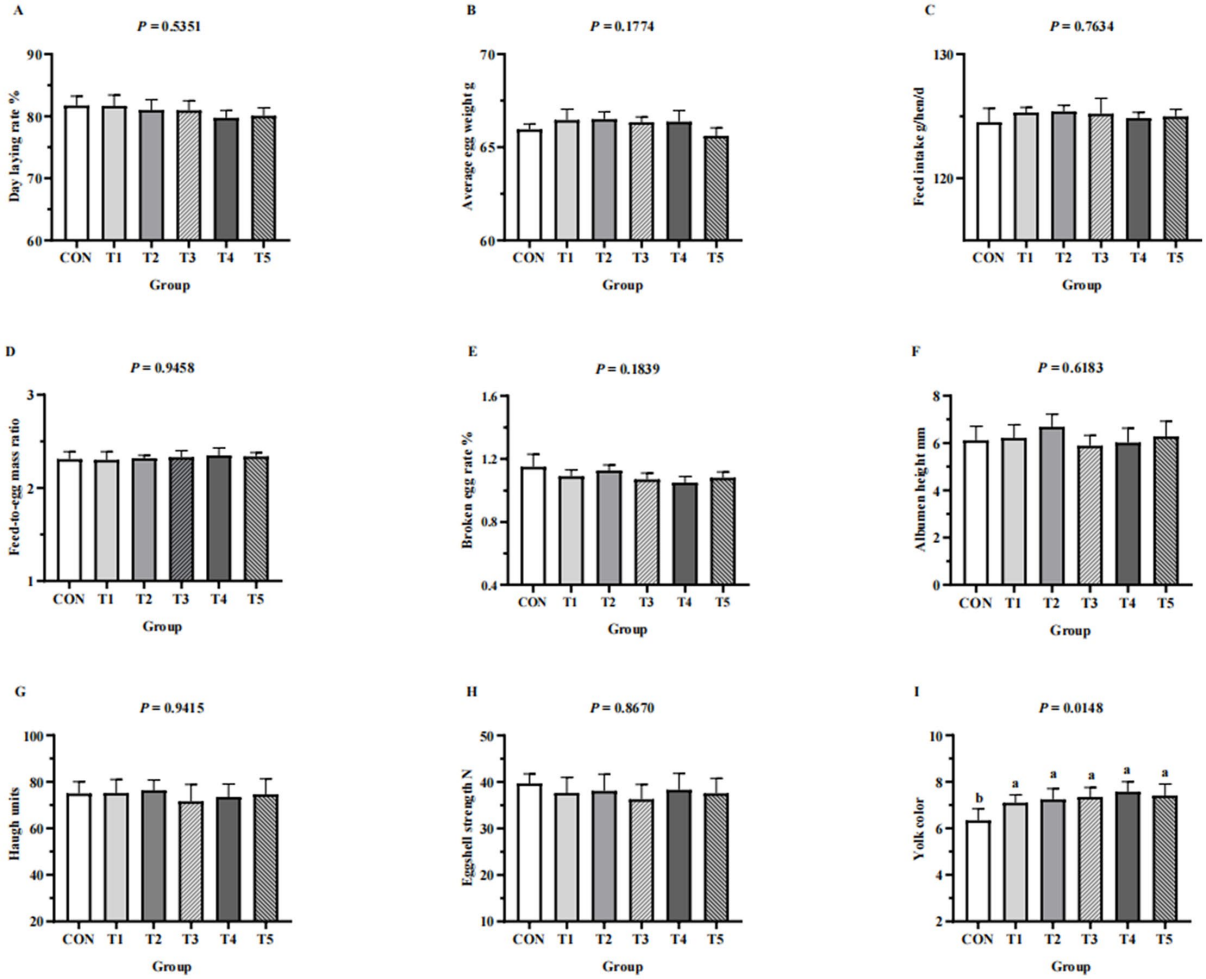
Effects of replacing DCP with MDCP to supplement P on laying performance and egg quality of Hy-Line Brown aged laying hens (69 to 78  weeks of age). **(A–E)** Laying performance; **(F–I)** egg quality. CON: DCP, with NPP supplementation at 0.20%. T1–T5: MDCP, with NPP supplementation at 0.07% (T1), 0.11% (T2), 0.15% (T3), 0.18% (T4), and 0.20% (T5). Phytase was used at 1470 FTU/kg diet. The statistical unit was each replicate (*n* = 5) for laying performance parameters and each egg (*n* = 50, with 10 eggs per replicate randomly selected) for egg quality parameters. ^a,b^Means within a row lacking a common superscript differ (*p* < 0.05).

Laying performance (day laying rate, average egg weight, feed intake, feed-to-egg mass ratio, broken egg rate) and egg quality (eggshell strength, albumen height, haugh units, yoke color) of hens from different groups are shown in Figure 1. Laying performance and egg quality (eggshell strength, albumen height, haugh units) were not affected by dietary supplementation with MDCP Pi (*p* > 0.05). The exception was yoke color, which improved in hens fed MDCP Pi compared with those fed DCP Pi (*p* = 0.0148).

### Serum biochemistry and serum Ca and P metabolism-related hormones

**Figure 2:**
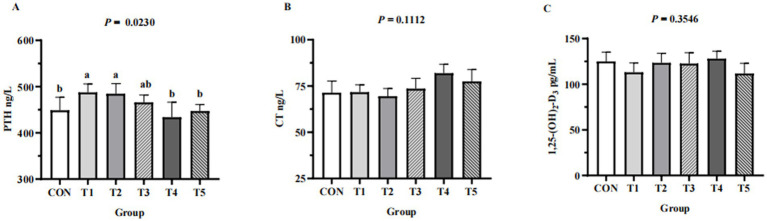
Effects of replacing DCP with MDCP to supplement P on serum Ca and P metabolism-related hormones of Hy-Line Brown aged laying hens (69 to 78  weeks of age). **(A)** PTH, parathyroid hormone; **(B)** CT, calcitonin; **(C)** 1,25-(OH)_2_D_3_, 1,25-dihydroxy-vitamin D3. CON: DCP, with NPP supplementation at 0.20%. T1–T5: MDCP, with NPP supplementation at 0.07% (T1), 0.11% (T2), 0.15% (T3), 0.18% (T4), and 0.20% (T5). ^a,b,c^ Means within a row lacking a common superscript differ (*p* < 0.05).

Across groups, no differences were detected in serum levels of Ca (*p* = 0.1,212), P (*p* = 0.8354), Cu (*p* = 0.6545), Fe (*p* = 0.8221), Zn (*p* = 0.8166), and Mn (*p* = 0.9557) (Table 4). Hens fed 0.07% and 0.11% NPP MDCP Pi had higher (*p* < 0.05) serum levels of PTH than those in all other groups (Figure 2). No differences were detected among groups in serum CT and 1,25-(OH)2D3 (*p* > 0.05).

**Table 4:** Effects of replacing DCP with MDCP to supplement P on serum biochemistry of Hy-Line Brown aged laying hens (69 to 78  weeks of age).^a^^,^^b^

Group							
Items	CON	T1	T2	T3	T4	T5	*p*-value
Ca (mmol/L)	4.64 ± 0.43	4.78 ± 0.15	4.28 ± 0.15	4.84 ± 0.64	3.98 ± 0.29	4.94 ± 0.62	0.1212
P (mmol/L)	1.50 ± 0.16	1.57 ± 0.11	1.56 ± 0.23	1.55 ± 0.16	1.68 ± 0.14	1.62 ± 0.19	0.8354
Cu (pmol/L)	33.55 ± 4.42	35.35 ± 1.84	33.97 ± 3. 13	32.28 ± 3. 12	31. 15 ± 2.25	34.20 ± 3.49	0.6545
Fe (pmol/L)	19.55 ± 1.40	19.58 ± 1.70	19.73 ± 2.60	20.47 ± 1. 10	19.01 ± 1.27	20.58 ± 0.86	0.8221
Zn (pmol/L)	57.70 ± 4.68	56.62 ± 5.70	61. 19 ± 6.73	58.60 ± 5.96	61.88 ± 3.78	61.53 ± 7.58	0.8166
Mn (pmol/L)	9.82 ± 1.34	9.81 ± 1. 10	9.95 ± 0.54	10.14 ± 0.84	9.90 ± 0.44	9.56 ± 0.92	0.9557

CON: DCP, with NPP supplementation at 0.20%.

T1–T5: MDCP, with NPP supplementation at 0.07% (T1), 0.11% (T2), 0.15% (T3), 0.18% (T4), and 0.20% (T5).

a Values are the mean ± standard error. Phytase was used at 1470 FTU/kg diet.

b The statistical unit was each hen (*n* = 10; at the end of feeding trial, with samples collected from two randomly selected laying hens per replicate).

### Tibia quality

The tibia breaking strength was significantly higher in the 0.18 and 0.20% NPP MDCP Pi groups than in the 0.20% NPP DCP Pi group (Table 5). The breaking strength, Ca content, and P content of tibia in 0.11% and 0.15% NPP MDCP Pi hens were not significantly (*p* > 0.05) different from those in 0.20% NPP DCP Pi hens (Table 5). In the 0.07% NPP MDCP group, the Ca and P contents of tibia were significantly lower (*p* < 0.05) than those in all other groups (Table 5).

**Table 5:** Effects of replacing DCP with MDCP to supplement P on tibia quality of Hy-Line Brown aged laying hens (69 to 78 weeks of age).^a^^,^^b^^,^^c^

Items	Group						*p*-value
	CON	T1	T2	T3 6	T4	T5 6	
Dry weight of degreased tibia (g)	6.70 ± 0.17	6.35 ± 0.31	5.89 ± 0.46	6.13 ± 0.56	6.09 ± 0.47	6.11 ± 0.49	0.2672
Breaking strength (kg/cm^2^)	15.41^c^ ± 1.89	16.45^bc^ ± 1.64	16.54^bc^ ± 2.2	16.76^bc^ ± 3. 19	18.43^ab^ ± 2.2	20.00^a^ ± 2.5	0.0051
Ash (%)	60.40^bc^ ± 0.42	59.98^c^ ± 0.32	0	60.27^c^ ± 0.36	9	8	0.0047
			60.04^c^ ± 0.64		61.10^a^ ± 0.73	61.05^ab^ ± 0.61	
Ca (%)	20.50^ab^ ± 0.28	19.86^c^ ± 0.23	20.27^b^ ± 0.26	20.57^ab^ ± 0.41	20.84^a^ ± 0.43	20.94^a^ ± 0.31	0.0003
P (%)	8.47^a^ ± 0.47	7.98^b^ ± 0.26	8.47^a^ ± 0.50	8.59^a^ ± 0.45	8.75^a^ ± 0.60	8.57^a^ ± 1.08	0.0255

CON: DCP, with NPP supplementation at 0.20%.

T1–T5: MDCP, with NPP supplementation at 0.07% (T1), 0.11% (T2), 0.15% (T3), 0.18% (T4), and 0.20% (T5).

a Values are the mean ± standard error. Phytase was used at 1470 FTU/kg diet.

b Means within a row with different superscript lowercase letters are different (*p* < 0.05).

c The statistical unit was each hen (*n* = 10; at the end of feeding trial, with samples collected from two randomly selected laying hens per replicate).

### Serum bone turnover markers

**Figure 3:**
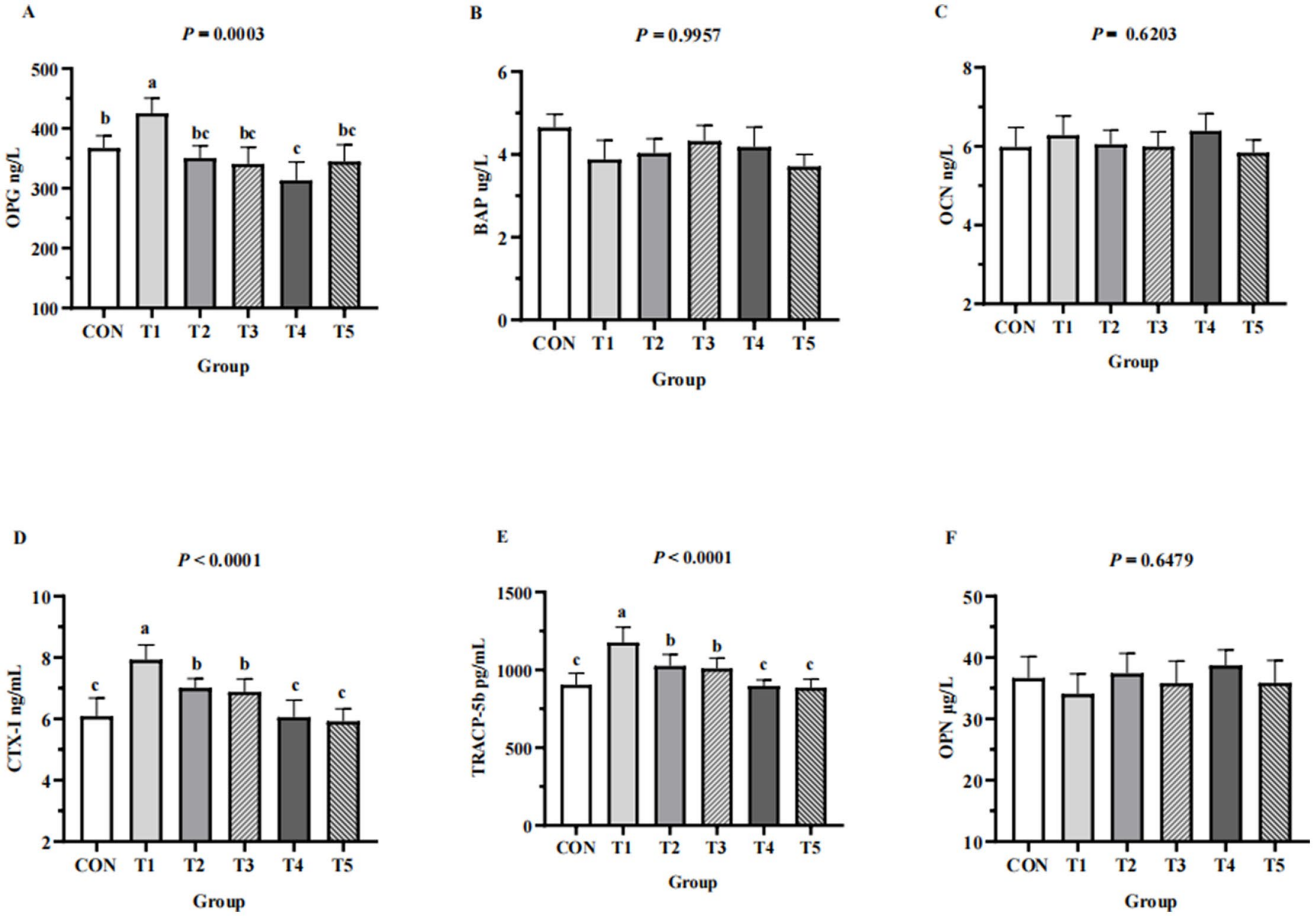
Effects of replacing DCP with MDCP to supplement P on serum bone turnover markers of Hy-Line Brown aged laying hens (69 to 78  weeks of age). CON: DCP, with NPP supplementation at 0.20%. T1–T5: MDCP, with NPP supplementation at 0.07% (T1), 0.11% (T2), 0.15% (T3), 0.18% (T4), and 0.20% (T5). **(A–C)** Serum bone formation markers; **(D–F)** Serum bone resorption markers. BAP, bone-alkaline phosphatase; OCN, osteocalcin; OPG, osteoprotegerin; CTX-I, C-telopeptide of I collagen; OPN, osteoponin; TRACP-5b, tartrate resistant acid phosphatase 5b. ^a,b,c^Means within a row lacking a common superscript differ (*p* < 0.05).

Serum bone formation markers (BAP, OCN, OPG) and serum bone resorption markers (CTX-I, OPN, TRACP-5b) of hens from different groups are shown in Figure 3. Serum levels of TRACP-5b and CTX-I in the 0.11% and 0.15% NPP MDCP Pi group were significantly lower than those in 0.15 to 0.20% NPP MDCP Pi groups and the 0.20% NPP DCP Pi group (*p* < 0.0001). Hens fed 0.07% NPP MDCP Pi had higher (*p* < 0.01) serum levels of OPG, CTX-I, and TRACP-5b than those in all other groups. Serum levels of OPG in the 0.11% NPP MDCP Pi group were not significantly different from those in 0.15 to 0.20% NPP MDCP Pi groups and the 0.20% NPP DCP Pi group (*p* > 0.05). No differences were detected in serum BAP, OCN, and OPN among groups (*p* > 0.05).

### Renal expression of NaPi-IIa

**Figure 4:**
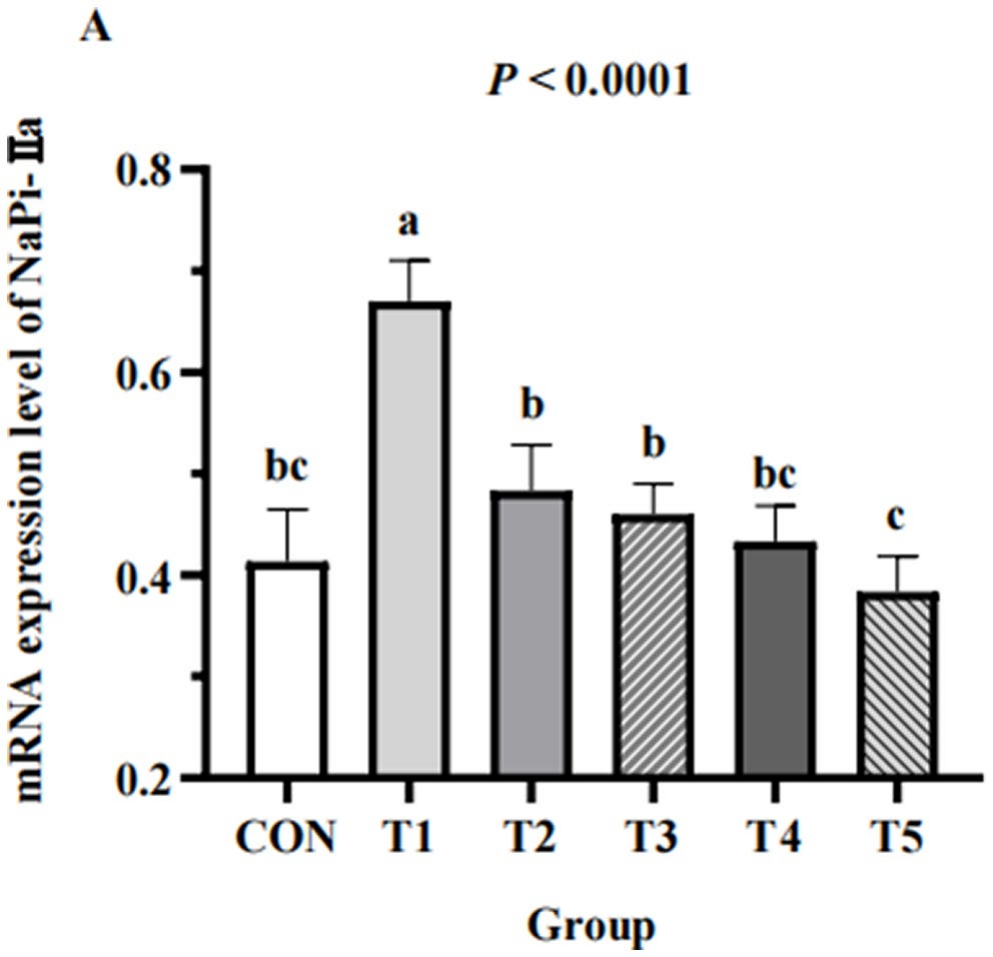
Effects of replacing DCP with MDCP to supplement P on expression of P transporters type IIa Na/Pi cotransporter (NaPi-IIa) in the kidneys of Hy-Line Brown aged laying hens (69 to 78  weeks of age). Relative mRNA expression was calculated for the housekeeping gene β-actin using the 2^−ΔΔCt^ method. ^a,b,c^ Means within a row lacking a common superscript differ (*p* < 0.05).

The expression of NaPi-IIa in the kidneys of hens from different groups are shown in Figure 4. The expression of NaPi-IIa in 0.11% and 0.15% NPP MDCP Pi hens were higher than those in 0.20% NPP MDCP Pi group and 0.20% NPP DCP Pi group (*p* < 0.05). Hens fed 0.07% MDCP Pi had the highest (*p* < 0.0001) renal expression of NaPi-IIa than those in all other groups.

## Discussion

Jing et al. (23) reported that reducing dietary NPP up to 0.15% is adequate to maintain health and performance of laying hens. Cheng et al. (24) reported that laying performance of Hy-Line Brown laying hens(29 to 40 weeks of age) was well maintained by diets supplemented with 2,000 FTU/kg phytase and 0.05%–0.15%NPP MDCP Pi (dietary NPP levels of 0.17–0.27%). Jing et al. (15) indicated that dietary NPP levels could be reduced to 0.10% with the inclusion of 1,000 FTU/kg phytase, without negative effects on production performance and health of the Lohmann hens from 22 to 34 weeks of age. In the current study, laying performance and egg quality were not significantly different among dietary treatments, indicating that 0.07%-0.15% NPP MDCP Pi with 1,470 FTU/kg phytase was sufficient to support the laying performance of Hy-Line Brown 69–78-week-old laying hens compared to 0.20% NPP DCP Pi. Because significant differences were not observed in egg weight and feed-to-egg mass ratio in the current trial, use of MDCP Pi could clearly reduce feed costs and environmental pollution by reducing the amount of Pi. However, the feeding trial was conducted in autumn, and the new corn was low in energy, resulting in an average daily hen intake of more than 120 g, which exceeded the standard of Hy-Line Brown-Conventional Systems in 2021. Given that limitation, long-term feeding studies with large numbers of hens are needed to confirm the findings of this study. There are large changes in P intake due to differences in feed intake.

Tibia quality is a crucial indicator of P nutrition in poultry (25), and inadequate or excess dietary P can cause bone disease (26, 27). Cheng et al. (24) reported that tibia P and Ca storage and mobilization were well maintained when diets were supplemented with 0.05%–0.15% NPP MDCP Pi (dietary NPP levels of 0.17–0.27%). Ren et al. (14) found that 0.20–0.30% DCP Pi (dietary NPP levels of 0.32–0.42%) did not significantly increase tibia breaking strength, ash content, or Ca and P contents in Hy-Line Brown 29–40-week-old laying hens. In the current study, compared with 0.20% NPP DCP Pi, tibia measurements were not affected in hens fed 0.11% and 0.15% NPP MDCP Pi. The supplementation with 0.11% and 0.15% NPP MDCP Pi (dietary NPP levels of 0.23 and 0.27%) is sufficient for the skeleton health of aged laying hens (69 to 78 weeks of age) with the presence of 1,470 FTU/kg phytase. In modern egg production, laying hens face a number of welfare problems related to the increased prevalence of fractures, especially a continuous high egg production in commercial layer lines have led to increased bone fragility and susceptibility to fracture (28, 29). Bone disease are detrimental to egg production, thereby negatively affecting egg quality and production performance (30). Still, minimal information is available on the effects of MDCP use on skeletal-related welfare problems in laying hens. In this study, the tibia breaking strength was significantly higher in the 0.18 and 0.20% NPP MDCP Pi groups than in the 0.20% NPP DCP Pi group, indicating that MDCP was more beneficial than DCP for tibia quality. Therefore, replacing DCP with MDCP to supplement P can be considered a positive welfare attribute.

Ca, P, and related hormones (PTH, CT and 1,25-(OH)_2_D_3_) in the serum are crucial indicators for phosphate–calcium metabolism. Jing et al. (23) reported that the concentrations of plasma P were generally reduced in hens on low-P diets. In this study, there were no significant differences in serum Ca and P in hens from different groups. The differences in findings could be related to different testing environments and sources of phytase (31). In addition, serum P are controlled by a variety of factors, and regulation is not limited to Pi intake alone but also to bone resorption and renal reabsorption (32). Meanwhile, the data from the current study showed that hens fed 0.07 and 0.11% NPP MDCP Pi had higher serum levels of PTH than those in all other groups. PTH, secreted from cells of the parathyroid glands, maintains the serum P levels at a normal level by acting directly or indirectly on the intestine, bone, and kidneys (33, 34).

The intake of P plays an important role in bone health, especially during egg laying. The effects of replacing DCP with MDCP and reducing NPP supplementation levels on several serum bone turnover markers indicating bone health were evaluated in this study. A biochemical marker of bone turnover secreted by osteoclasts, TRACP-5b reflects the status of bone resorption, and its blood concentration is used to detect the total amount of bone resorption (25). Obrant et al. (35) reported that serum TRACP-5b is significantly elevated in women with persistent fractures. CTX-I is a special sequence fragments released by osteoclasts from the degradation of type I collagen during bone resorption (36). Therefore, CTX-I reflects the level of bone destruction and is a specific indicator of bone resorption (37). OPG is an irreducible soluble receptor secreted by osteoblasts, which is considered as a protective factor against bone loss (38, 39). OPG could regulate the differentiation of osteocytes and block the formation of osteoclasts (1). Khosla et al. (40) reported a compensatory increase in OPG inhibition of abnormal bone resorption in postnatal rats due to lactation, which disturbed Ca and P metabolism *in vivo*. The results showed that the serum levels of OPG in the 0.07% NPP MDCP Pi group increased significantly, indicating that the speed of bone resorption was accelerated, which resulted in abnormal bone loss (41). This conclusion is consistent with the lowest tibia Ca and P contents in the 0.07% NPP MDCP group. Aged hens may be more sensitive to P deficiency than young chicks (42). In the current study, the serum levels of TRACP-5b and CTX-I in the 0.11% and 0.15% NPP MDCP Pi hens were significantly lower than those in 0.18 and 0.20% NPP MDCP Pi and 0.20% NPP DCP Pi hens. Low-P intake caused hens to mobilize bone resorption to maintain the body’s calcium-phosphorus metabolism balance.

In addition to the above biochemical indices, the related mRNA expression of a P transporter was also examined. The type II sodium/phosphate cotransporter (NaPi-II) group primarily includes cotransporters NaPi-IIa, NaPi-IIb, and NaPi-IIc, with NaPi-IIb mainly in the small intestine and NaPi-IIa and NaPi-IIc mediating renal P reabsorption (43). The P-transport capacity of the kidney is the most important factor in the maintenance of P homeostasis in hens (44). In a kidney, NaPi-IIa is an electrogenic transporter that preferentially combines with P, whereas NaPi-IIc is an electroneutral transporter that does not easily combine with P. Dietary P deficiency induces upregulation of renal NaPi-IIa mRNA expression in poultry (45). Katsumata et al. (27) reported a significant decrease in renal NaPi-IIa mRNA expression levels after feeding a high-P diet to rats. Li et al. (46) reported that laying hens fed 0.15% available phosphoru (AP) had higher NaPi-IIa mRNA expression levels than those fed 0.41–0.82% AP. In the current study, the renal NaPi-IIa mRNA expression level in the 0.07%–0.15% NPP hens was higher than that in 0.18–0.20% NPP hens, which indicated that the low-P diet increased renal P reabsorption. Because there are no antibodies to NaPi-IIa, protein expression of NaPi-IIa in the kidney was not measured in the current study. Therefore, the effect of a low-P diet on phosphorus balance in laying hens remains to be elucidated.

## Conclusion

Renal P reabsorption and bone resorption were both involved in adapting to a low-P diet. In conclusion, NPP levels can be reduced to 0.11% (dietary NPP level of 0.23%) when MDCP is used to supplement NPP in diets instead of DCP without negatively affecting laying performance and skeletal health of aged hens.

The nutritional value of different P sources should be reconsidered in laying hens, especially in the application of phytase. There is great potential to reduce feed costs and decrease phosphorus excretion by reducing the amount of inorganic phosphates without affecting skeletal health and performance of aged hens.

## Data availability statement

The original contributions presented in the study are included in the article/supplementary material, further inquiries can be directed to the corresponding authors.

## Ethics statement

The animal study was reviewed and approved by The Animal Care and Use Committee of Shandong Agriculture University (protocol code SDAUA-2021-019).

## Author contributions

WY and SJ received funding. YR, TZ, NJ, and YL conceptualized and designed the study. YR, TZ, LL, ZZ, and KZ conducted animal experiments, chemical analyses, and analyzed the data. YR and TZ wrote the original. NJ, SJ, and YL reviewed and revised the draft. All authors contributed to the article and approved the submitted version.

## Funding

This research was supported by the Major Innovative Projects of Shandong Province (2019JZZY020609). WY and SJ received funding.

## Conflict of interest

The authors declare that the research was conducted in the absence of any commercial or financial relationships that could be construed as a potential conflict of interest.

## Publisher’s note

All claims expressed in this article are solely those of the authors and do not necessarily represent those of their affiliated organizations, or those of the publisher, the editors and the reviewers. Any product that may be evaluated in this article, or claim that may be made by its manufacturer, is not guaranteed or endorsed by the publisher.
